# Natural Time Analysis of Seismicity within the Mexican Flat Slab before the M7.1 Earthquake on 19 September 2017

**DOI:** 10.3390/e22070730

**Published:** 2020-06-30

**Authors:** E. Leticia Flores-Márquez, Alejandro Ramírez-Rojas, Jennifer Perez-Oregon, N. V. Sarlis, E. S. Skordas, P. A. Varotsos

**Affiliations:** 1 Instituto de Geofísica, Universidad Nacional Autónoma de México, Circuito Institutos S/N, C.U., C.P. 04510 México City, Mexico; 2 Departamento de Ciencias Básicas, Universidad Autónoma Metropolitana, C.P. 14387 México City, Mexico; alexramro@gmail.com; 3 Departamento de Física, Escuela Superior de Física y Matemáticas, Instituto Politécnico Nacional, UP Zacatenco, C.P. 07738 México City, Mexico; jnnfr.po@gmail.com; 4 Department of Physics, Solid Earth Physics Institute, National and Kapodistrian University of Athens, Panepistimiopolis, 157 84 Zografos, Greece; nsarlis@phys.uoa.gr (N.V.S.); eskordas@phys.uoa.gr (E.S.S.); pvaro@otenet.gr (P.A.V.); 5 Section of Condensed Matter Physics, Department of Physics, National and Kapodistrian University of Athens, Panepistimiopolis, 157 84 Zografos, Greece

**Keywords:** seismicity, natural time analysis, entropy, variability, nowcasting, plate tectonics

## Abstract

One of the most important subduction zones in the world is located in the Mexican Pacific Coast, where the Cocos plate inserts beneath the North American plate. One part of it is located in the Mexican Pacific Coast, where the Cocos plate inserts beneath the North American plate with different dip angles, showing important seismicity. Under the central Mexican area, such a dip angle becomes practically horizontal and such an area is known as flat slab. An earthquake of magnitude M7.1 occurred on 19 September 2017, the epicenter of which was located in this flat slab. It caused important human and material losses of urban communities including a large area of Mexico City. The seismicity recorded in the flat slab region is analyzed here in natural time from 1995 until the occurrence of this M7.1 earthquake in 2017 by studying the entropy change under time reversal and the variability *β* of the order parameter of seismicity as well as characterize the risk of an impending earthquake by applying the nowcasting method. The entropy change Δ*S* under time reversal minimizes on 21 June 2017 that is almost one week after the observation of such a minimum in the Chiapas region where a magnitude M8.2 earthquake took place on 7 September 2017 being Mexico’s largest quake in more than a century. A minimum of *β* was also observed during the period February–March 2017. Moreover, we show that, after the minimum of Δ*S*, the order parameter of seismicity starts diminishing, thus approaching gradually the critical value 0.070 around the end of August and the beginning of September 2017, which signals that a strong earthquake is anticipated shortly in the flat slab.

## 1. Introduction

The earthquakes (EQs) occur principally between subduction plates or faults. A tectonic consequence of the subduction process is the occurrence of inter-plate and intra-plate EQs where the Wadati–Benioff zones are defined [1,2]. Uyeda and Kanamori [2], classified in 1979 the subduction zones in the Marianas-type and Peru-Chile type. The Marianas-type is characterized because its subduction angle is almost vertical and conformed by an old oceanic plate, whereas the second one has a fast and younger plate moving with a soft subduction angle attaining some regions as horizontal plateau named flat slab.

**Figure 1:**
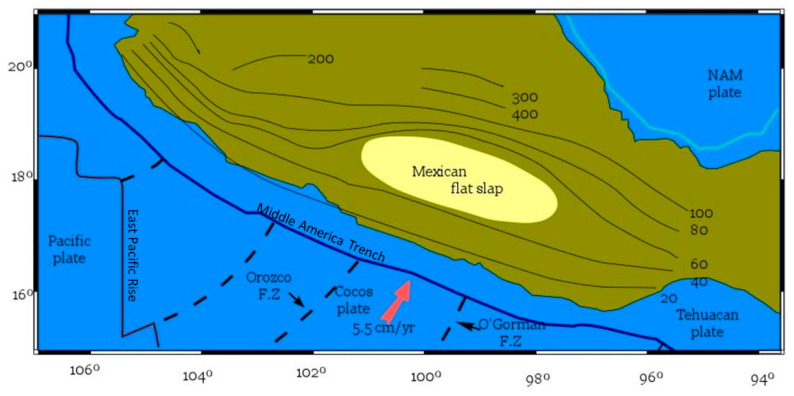
Map of the Mexican flat slab. The isolines of Moho depths have been drawn in accordance to those reported in Reference [1].

This kind of subduction has been observed, principally in three sites in the world, Central Chile, Peru, and Central Mexico (see Figure 1 in Reference [1]), each of which however has its own characteristics. The flat slab of the Mexican subduction zone is of major interest, historically EQs inland have struck big cities like the EQ that occurred on 19 September 2017, close to Mexico City, causing important human losses and significant material damages around downtown area. The Mexican flat slab area is located along the central part of the Cocos and North American plates.

The EQ on 19 September 2017, occurred 32 years after the great EQ that struck the Mexico City in 1985 and on the same month and day, happened in Michoacán State, in the subduction zone between the Cocos and North American plates. It also happened 12 days after the M8.2 EQ in Chiapas, on Tehuantepec Gulf, within the Cocos plate itself which is the largest earthquake in Mexico for more than a century. To summarize, the two quakes occurred in the same year 2017, at two different spots in the Cocos tectonic plate, in the Mexican subduction zone, and the M7.1 EQ on 19 September 2017 occurred near the northern limit of the Mexican flat slab, which represents an important seismically active zone in central Mexican region, as already mentioned. Here, we investigate possible precursory phenomena of seismicity that appeared before the latter EQ, while such phenomena associated with the former EQ (i.e., the M8.2 on 7 September 2017) have already discussed elsewhere [3,4,5,6].

Several methods have been used to study the seismicity among which one can list the spectral analysis [7,8], complex EQ networks [9,10,11], entropy-based methods [3,12,13,14], Detrended Fluctuation Analysis (DFA) and multifractal analysis [15,16], Allan factor [17,18], Higuchi fractal dimension [19,20], and natural time analysis, see Reference [21] and references therein (see below). For instance, Ramírez-Rojas et al. [7] estimated the temporal correlations calculating the spectral analysis of geoelectric time series monitored in the south Pacific Mexican coast and several months before the M6.4 EQ on 24 September, 1994. The study showed long-range correlations since some months before the main shock, and after that, the correlations disappeared suggesting that the preparation stage evolved to attain a critical state [21], being the main shock like a phase transition. To study such a transition for seismicity, an order parameter must be defined.

An appropriate order parameter denoted $\kappa_{1}$ has been introduced [21,22,23] in natural time analysis, which allows us to identify when the system approaches a critical state, [21]. This has been obtained for several dynamical models (see Chapter 8 of Reference [21], see also Reference [23]) as well as for several mainshock occurrences, when $\kappa_{1}$ approaches the value 0.070. Another physical quantity defined in natural time analysis is the entropy change Δ*S* under time reversal [24] which help us to uncover hidden features in complex systems time series of as for example to identify the approach of a dynamic phase transition [25].

Rundle et al. [26], see also, e.g., References [14,27,28,29,30] proposed a methodology recently known as “nowcasting” to estimate the seismic risk level based on natural time.

In the present paper, the seismic activity in the Mexican flat slab region is studied in natural time since 1995 until the occurrence of the M7.1 EQ on 19 September 2017. We will also introduce the most important tectonic aspects of the flat slab region, since this is the trigger for the great seismicity that occurs in the area. Results will be obtained for the entropy change under time reversal and the variability of the seismicity order parameter together with a procedure to estimate the date of the impending mainshock. Finally, we will also present the nowcasting results after applying this methodology just before the M7.1 EQ on 19 September 2017.

## 2. Methodology

### 2.1. Natural Time Analysis

Natural time analysis is based on a new definition of time introduced in Reference [22] (see Preface and Chapter 2 of Reference [21] and in particular its Section 2.1 and Section 2.2, as well as Reference [31]) and has been found of usefulness [21] to uncover important features hidden in complex systems time series spanning various disciplines from cardiology [25,32,33] to seismology (including laboratory fracture experiments under well controlled conditions) [3,4,5,6,34,35,36,37,38] and from atmospheric sciences [39,40] to complex networks [41], and civil engineering [42].

For a time series consisting of *N* events, the index for the occurrence of the *k*-th event given by χk=kN, is termed natural time. In this analysis, the elapsed time between consecutive events is ignored, but preserving the occurrence order and their energy *Q_k_*. For seismic catalogues $Q_{k\;}\propto \;10^{1.5M}$, where the moment magnitude [43] *M* is used [37,38,44]. In natural time we study the evolution of the pair ($\chi_{k}$, $Q_{k}$) or alternatively ($\chi_{k}$, $p_{k}$) where pk=Qk∑k=1NQk is the normalized energy for the *k*-th event. The normalized power spectrum is defined as $\Pi \left(\omega \right)={\left|\Phi \left(\omega \right)\right|}^{2}$ where $\Phi \left(\omega \right)=\sum_{k=1}^{N}p_{k}exp\left(i\omega \chi_{k}\right)$ and *ω* stands for the angular natural frequency. Note that $\chi_{k}$ is “rescaled” as natural time changes from *N* events to (*N* + 1) events as $\chi_{k\;}=k/\left(N+1\right)$ together with pk=Qk∑k=1N+1Qk upon the occurrence of any new event.

The behavior of Π(ω) is studied when ω approaches zero, since all the statistical moments of the distribution of the *p_k_*, can be determined from *Π(ω)* in the limit ω→0 (see page 130 in Reference [21]). From the Taylor expansion of Π(ω) the quantity $\kappa_{1}$ is defined as:

(1) $$\Pi \left(\omega \right)=1-\kappa_{1}\omega^{2}+\kappa_{2}\omega^{4}+\ldots ,$$

where:

(2) $$\kappa_{1}=\overset{N}{\underset{k=1}{\sum }}p_{k}\chi_{k}^{2}-{\left(\overset{N}{\underset{k=1}{\sum }}p_{k}\chi_{k}\right)}^{2}$$

This is the variance $\kappa_{1}=\langle \chi^{2}\rangle -\langle \chi^{2}\rangle$, and has played an interesting role as a key parameter when analyzing seismic catalogues [5,37,38,41]. This quantity, $\kappa_{1}$, is very important in view of the following: It is generally accepted [21,45,46] that EQs, which show complex correlations in time, space and magnitude (e.g., [47,48,49,50,51,52,53,54]), can be regarded as critical phenomena where the mainshock is the new phase. The parameter $\kappa_{1}$, as shown in detail in Reference [23], is the order parameter of seismicity by means of which one can determine when the system approaches to the critical point.

The entropy in natural time domain, *S*, is given by [55]:

(3) S=〈χlnχ〉−〈χ〉ln〈χ〉

where the bracket refers to the expected value $\langle f\left(x\right)\rangle =\sum_{k=1}^{N}p_{k}f\left(x_{k}\right)$. It is a dynamic entropy showing [24] concavity, positivity and Lesche stability [56,57] and its value *S_u_* in a uniform (*u*) distribution [21] is *S_u_* = 0.096 (for its dependence on *N* see Reference [24] and its Supplementary Information as well as Section 3 of Reference [21]). Applying the time reversal operator $\hat{T}p_{k}=p_{N-k+1}$ to the entropy, the entropy under time reversal, *S*_, is obtained from:

(4) $$S\_\;=\;\overset{N}{\underset{k=1}{\sum }}P_{N-k+1}\left(\frac{k}{N}\right)\ln\left(\frac{k}{N}\right)-\left(\overset{N}{\underset{k=1}{\sum }}P_{N-k+1}\left(\frac{k}{N}\right)\right)\ln\left(\overset{N}{\underset{k=1}{\sum }}P_{N-k+1}\left(\frac{k}{N}\right)\right)$$

It is clear that *S* and *S*_ behave differently so that the difference, Δ*S* = *S* − *S*_, represents an important parameter, whose physical meaning has been studied [58] by means of the probability distribution function *P*(*χ*;∈) = 1 + ∈(*χ* − 1/2) defined for *χ* ∈ (0,1] instead of the discrete distribution $p_{k}$. In Reference [21] (see page 183) was shown that for small ∈ Δ*S*(∈) = ((6 ln 2 − 5)/36) ∈ + *O*($\in^{3}$) which results in negative Δ*S* for an increasing (∈>0) trend.

Δ*S* is a key measure [21] which may determine the approach to a dynamic phase transition. There are some examples where Δ*S* was employed [25] for the determination of the approach to sudden cardiac death. The estimation of complexity measures [4,21,32] based on Δ*S* has been of great importance to investigate the predictability [59] of the Olami-Feder-Christensen (OFC) EQ model [60], which is one of the most studied [61] non-conservative, supposedly, self-organized criticality (SOC) model [62]. OFC was originated as a simplification of the Burridge–Knopoff spring-block model [63]. In Reference [59] was shown that the value of *S*_ − *S* exhibits a clear maximum, thus Δ*S*(= *S* − *S*_) is minimum [21], before strong avalanches in the OFC model, thus this minimum points to an impending strong avalanche corresponding to a strong EQ.

For time series of *N* events, usually the calculation of entropy and the entropy under time reversal are performed with a moving window comprising a number i of consecutive events, which for reasons of brevity will be also called scale, and Δ*S* is denoted with a subscript i, as (Δ*S_i_*).

As for, the variability $\beta_{i}$ of the order parameter $\kappa_{1}$, [21], this is defined as follows: Considering a sliding natural time window consisting of *i* successive events moving, event by event, through the EQ catalogue, the calculated $\kappa_{1}$ values enable the estimation of their average value $\mu \left(\kappa_{1}\right)$ and their standard deviation $\sigma \left(\kappa_{1}\right)$. The quantity $\beta_{i}$ [64]:

(5) $$\beta_{i}=\frac{\sigma \left(\kappa_{1}\right)}{\mu \left(\kappa_{1}\right)}$$

Corresponding to this window of length i is called variability of $\kappa_{1}$ and its time evolution $\beta_{i}$ is followed by using the procedure of References [65,66]: First, we consider an excerpt consisting of i consecutive EQs from the Mexican flat slab seismic catalogue with *M* ≥ 3.5. We then form its sub-excerpts comprising the *n*-th to the (*n* + 5)-th EQs, (*n* = 1, 2, …, i − 5) and calculate $\kappa_{1}$ for each of them. By doing this, we set $\chi_{k}=k/6$ and $p_{k}=Q_{k}/\sum_{n=1}^{6}Q_{n}$, *k* = 1, 2, …, 6 to the *k*-th member of each sub-excerpt (cf. at least 6 EQs are needed for obtaining a reliable $\kappa_{1}$ [23]). We iterate this process for new sub-excerpts consisting of 7 EQs, 8 EQs, …, and finally i EQs. Then, we calculate the average $\mu \left(\kappa_{1}\right)$ and the standard deviation $\;\sigma \left(\kappa_{1}\right)$ of the thus obtained (i − 4)( i − 5)/2 $\kappa_{1}$ values. The variability $\beta_{i}$ for this excerpt i resulting from Equation (5) is assigned to the next EQ of the flat slab catalogue, which is called target EQ. The $\beta_{i}$ time evolution can be pursued by moving the window through the EQ catalogue and assigning $\beta_{i}$ to the occurrence date of the target EQ. The fluctuations of the order parameter of seismicity exhibit [67] a minimum $\beta_{min}$ upon the observation of a Seismic Electric Signals (SES) activity [68,69] which is precursory of a strong EQ. Once an SES activity has been initiated, a few weeks to $5\frac{1}{2}$ months before a strong EQ [21], the future epicentral area can be estimated by means of an SES selectivity map [68,69]. When electrical data are lacking, we rely on the following result [66]: A spatiotemporal study of $\beta_{min}$ unveils the future epicentral area.

### 2.2. Nowcasting

Nowcasting describes the present state of a system [26] and differs from forecasting, which looks forward in time [70,71,72,73].

Nowcasting, introduced in Reference [26], is an EQ method to determine the current hazard level in an active seismically region by counting the number of small EQs that occurred within the elapsed time between two large EQs within a defined region. In nowcasting Rundle et al. [26] measure the progress of the EQ cycle by using natural time event counts of small EQs between two large EQs. This is so because among the advantages of the application of natural time to seismicity are [26]: first, there is no need to decluster the EQ catalogue and second, only the natural interevent count statistics are used instead of the seismicity rate, which additionally demands calendar time. 

The implementation proposed by Rundle et al. [26] has found useful applications [14,27,28,29,30] and requires as principal information source a global catalogue of EQs. The nowcasting procedure considers the “large” EQs which have magnitude *M* ≥ *M_λ_*, where *M_λ_* denotes the “large” EQ threshold, and the “small” EQs, whose magnitude *M* is smaller than *M_λ_* but satisfies the condition *M* ≥ *M_σ_*. The threshold *M_λ_* is chosen to secure enough EQ cycles to provide reasonable statistics, e.g., at least ~20 or more large EQ cycles [26]. The small EQ magnitude threshold *M_σ_* is typically set by the catalogue completeness level. 

If we denote by $N_{c\sigma }$ the number of small EQs occurring between two large EQs, we can construct its cumulative distribution function $P\left(N_{c\sigma }\right)$ by tabulating $N_{c\sigma }$ and using standard methods (e.g., [74]). Since Gutenberg–Richter statistics are a good approximation and EQs exhibit [27] the ergodic property, the natural time count *n_s_* of small EQs since the last large EQ, should be a measure of the hazard for the next EQ with $M\geq M_{\lambda }$. The EQ potential score (EPS) for a large EQ to occur having magnitude larger than $M_{\lambda }$, is obtained by calculating the cumulative distribution function $P(N_{c\sigma }<n_{s})$.

## 3. Tectonic Subduction Structure

The Mexican subduction zone has been characterized as atypical since the Meso-American Subduction Experiment showed that subduction in southern Mexico is different from other subduction zones, where the large EQs occur in the so-called “Benioff zone”, at depths ranging from the Earth’s surface to about 600 km (http://web.gps.caltech.edu), and the majority of EQs in southern Mexico, occur at depths 0 to 50 km [75] and close to the coast. 

In Mexico, the Cocos plate is shaped in triangular form, bordered by the North American plate to the northeast, with the Caribbean plate to the southeast, and to the west by the Pacific plate. The flat slab subduction in western Mexico refers to the shallow dipping lower plate, occurring just at 10% of subduction zones. The present flat slab area is located along the central part of the Cocos-North America plate boundary that the convergence rate between Cocos and North America and the plate age increases only slightly to the southeast along the Middle America Trench (MAT) [76,77], the dip of the subducting slab varies strongly, from steep to flat [1]. In Central Mexico, according to Reference [78], between depths of 60–80 km, the exothermic phase transition in the subducting oceanic crust takes place.

In Reference [79], it was shown that the subducted Cocos plate beneath central Mexico becomes almost perfectly horizontal or flat at approximately 75 km from the MAT and around 50 km depth, running flat for approximately 175 km then in plunges steeply at ~75° into the mantle.

Manea et al. [1] presented a review of the tectonic dynamic evolution, where the tectonic plates velocities were estimated by means of the Indo-Atlantic hotspot reference frame [80] in order to determine the convergence rate velocities in the range 5–6 cm/y, for ~10 to 18 Ma, respectively. They pointed out that the flat slab runs almost perfectly and horizontal at ~45 km depth, of about 300 km inland from the MAT before sinking at a fairly steep angle of ~75° into the asthenosphere [81]. The Cocos plate contains a series of well-defined oceanic fracture zones (cf. the Orozco, O′ Gorman, and distant from the flat slab area and farther south, the Tehuantepec fracture zone) created by the physical extension of transform faults between offset spreading centers along the East Pacific Rise. Between the Orozco and the O′Gorman fracture zones, offshore the flat slab area, the oceanic plate surface is rather smooth (Figure 1) compared with the rugged surface of the neighboring regions [82]. The subduction geometry of the flat slab is important to understand its long-term geodynamic and tectonic evolution [83]. Some studies identified that the Mexican subduction zone presents large dip variations along strike [84,85], but in Reference [86] was also revealed that the Mexican flat slab lacks widespread EQs in both the fore-arc region and within the subducting Cocos slab.

In addition to these tectonic assessments, the flat slab has been shown important seismic activity, nonetheless it is less than in other seismic areas of Mexico. The flat slab was the region where the strong EQ on 19 September 2017, shook the Mexico City causing, as mentioned, dead and great economic losses. Mexican flat slab represents an important seismically active zone in central Mexican region.

## 4. Data and Analysis

**Figure 2:**
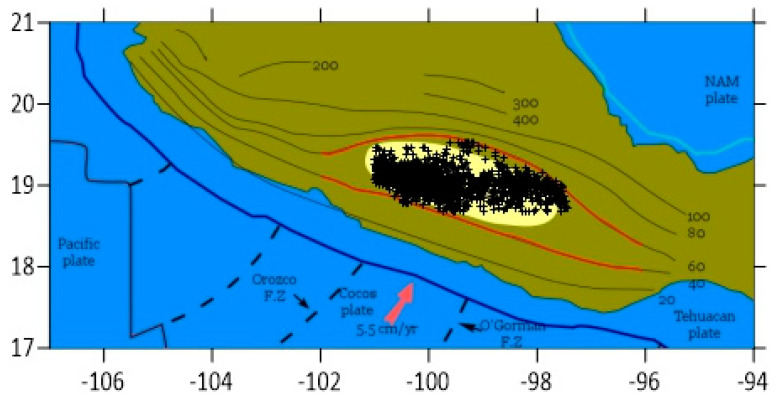

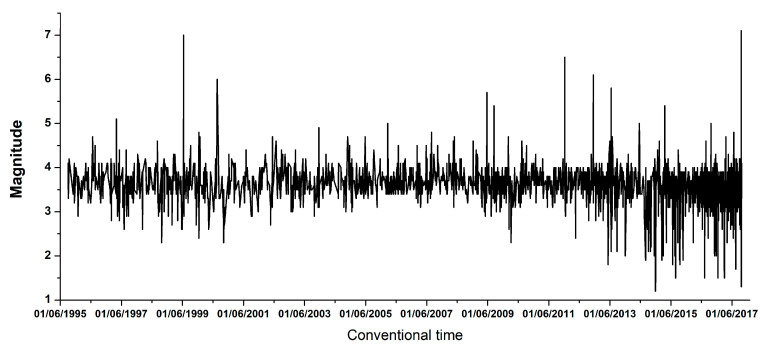
Seismicity in the flat slab from 1 September, 1995 to 24 September 2017. **Top** spatial distribution, **bottom** time distribution.

The EQ catalogue of the National Seismic Service (SSN) of the Universidad Nacional Autónoma de México UNAM (www.ssn.unam.mx) from 1 September, 1995 until 24 September 2017, was used here. Considering the area of the flat slab and taking just the EQs with epicenters situated between 40 and 60 km of Moho depths we plot their spatial distribution in the upper panel of Figure 2. The lower panel of this figure depicts their time distribution by plotting their magnitudes versus the conventional time of their occurrence. To assure catalogue completeness a magnitude threshold *M_σ_* = 3.5 has been imposed after studying the cumulative frequency magnitude distribution.

## 5. Results

### 5.1. Entropy in Natural Time Domain

The catalogue has registered 2137 EQs with *M* ≥ 3.0 and 1604 EQs with *M* ≥ 3.5 in the considered period (22 years), which is very low compared with approximately 11,500 EQs with *M* ≥ 3.5 in the period 2012–2017 monitored in the South Pacific coast.

**Figure 3:**
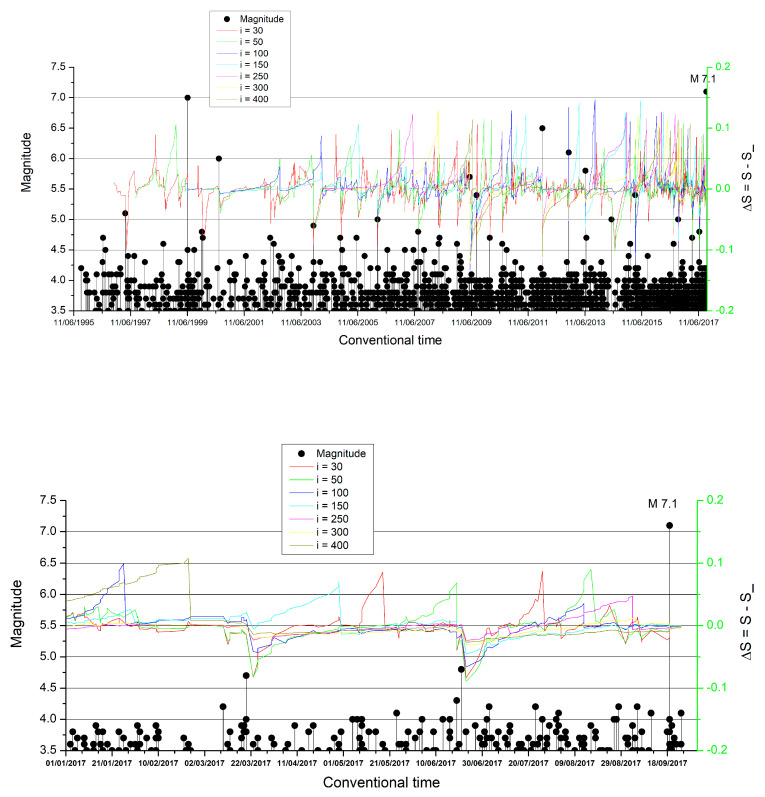
**Upper panel**: Δ*S_i_* = *S_i_* − *S_i__* versus the conventional time in the flat slab with windows (scales): i = 30, 50, 100, 150, 250, 300, and 400 consecutive events (*M* ≥ 3.5) for the whole catalogue from 1 January, 1995 until the M7.1 EQ occurrence on 19 September 2017. In the **lower panel**, ΔS is plotted for the last period before the main shock, i.e., from 1 January 2017 until the M7.1 EQ. It can be observed that Δ*S_i_* is attaining the minimum on 21 June, almost three months before the 19 September M7.1 EQ.

The entropy *S*, entropy under time reversal *S*_ and their difference Δ**S* = *S* − *S*_* were calculated by using several scales i. The selection of the minimum scale i was based on the aspects discussed in Reference [87] (see also References [12,88]), according to which the crucial scale should be in agreement with the number of EQs with magnitude *M* ≥ 3.5 that take place during an interval at least around the SES activities’ maximum lead time which is $5\frac{1}{2}$ months, as mentioned. Thus, since we have in total 1604 EQs *M* ≥ 3.5 for a period of 22 years, we find around i=30 events during $5\frac{1}{2}$ months (cf. the actual number is 33 which is approximated by 30). For example, in Figure 3 (upper panel) we depict the $\Delta S_{i}$ values versus the conventional time for the following scales: i = 30, 50, 100, 150, 250, 300, and 400 events. To better visualize what happened after the beginning of 2017, we plot in Figure 3 (lower panel) the $\Delta S_{i}$ values versus conventional time from 1 January 2017 until the M7.1 mainshock occurrence on 19 September 2017. 

An inspection of this figure reveals that $\Delta S_{i}$ exhibits a minimum upon the occurrence of a M4.8 EQ on 21 June 2017, i.e., approximately three months before the deadly M7.1 EQ. Remarkably, a similar minimum also appeared in the Chiapas area almost one week earlier, i.e., on 14 June 2017, upon computing, however, the $\Delta S_{i}$ values of seismicity in this area, where the 7 September 2017, M8.2 EQ took place as mentioned in Reference [3]. The appearance of the minimum on 21 June 2017 is statistically significant especially for ∆*S*_300_ and ∆*S*_400_ which simultaneously exhibit their deepest minimum since 28 November 2012 (an almost 5-year period) and correspond to the two longer scales, i.e., *i* = 300 and 400 EQs, respectively. Taking the view that EQ catalogues can be considered as marked point-processes [89,90] in which the times of EQ occurrences are marked by the EQ magnitudes, we randomly shuffled the marks during the last ten years of the EQ catalogue under study and constructed 10^2^ synthetic EQ catalogues for the flat slab. We found that only in 2% of the cases the deepest minima since 28 November 2012 of the ∆*S*_300_ and ∆*S*_400_ have been simultaneously observed up to one month after the ∆*S_i_* minimum identified on 14 June 2017 in the Chiapas area.

### 5.2. Variability Analysis

**Figure 4:**
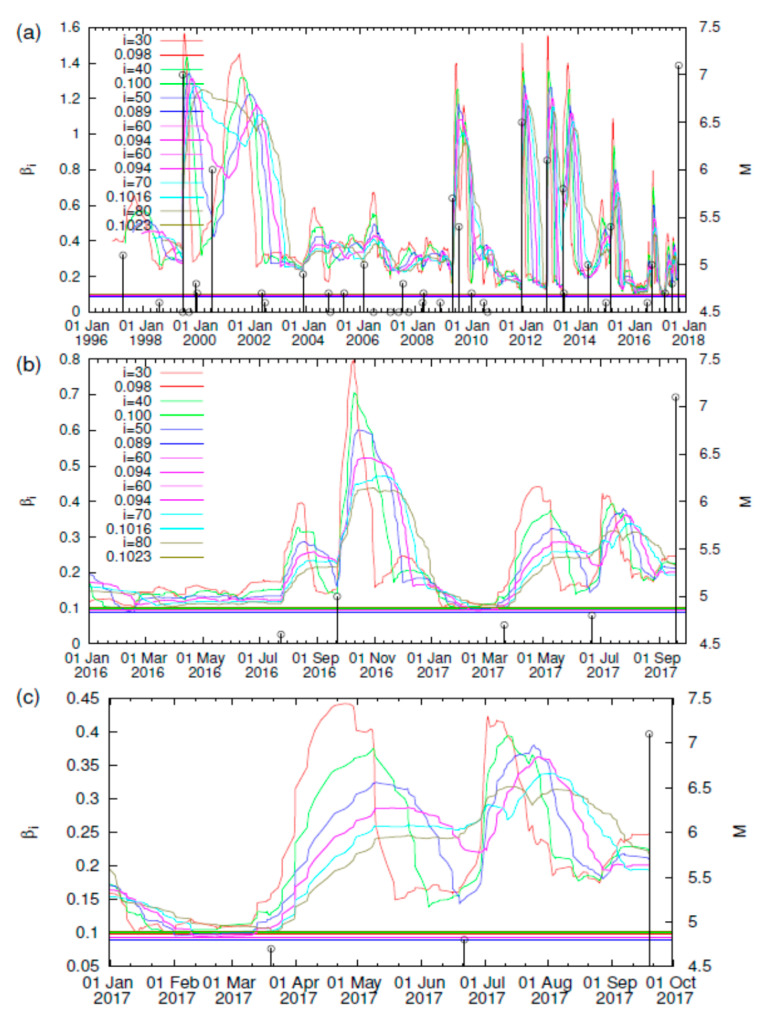
(**a**) The variabilities $\beta_{i}$ for i = 30, 40, 50, 60, 70, and 80 of the order parameter of seismicity *κ*_1_ in the Mexican flat slab during the whole 22-year period studied. Panel (**b**) is an excerpt of (**a**) but in expanded time scale. The horizontal lines correspond to the global minima for whole period studied. Panel (**c**) focuses on the smaller values of the variabilities $\beta_{i}$ in order to show the minima observed during the period February to March 2017 for i = 30, …, 80.

For reasons explained in the previous subsection the window values (or scales) around 30 events or larger have been used. In particular, our calculation was made for the following values: i.e., i = 30, 40, 50, …, 80 events and the results are depicted in Figure 4. We find that for i = 30, 40, 60, 70, and 80 a minimum is observed during the period February to March 2017, i.e., several months before the M7.1 EQ. Note that for i = 50 events, the global minimum appears during February 2016 with a value 0.089, but the minimum value attained during February 2017 is 0.096 which is the next deeper local minimum. Such minima in EQ catalogues have been shown to be statistical significant EQ precursors by various techniques like Monte-Carlo [91], random shuffling of EQ magnitudes [92], Receiver Operating Characteristics (ROC) [91], area under the ROC curve [93] and event coincidence analysis [38]. Thus, it appears that a $\;\beta_{i}\;$ minimum is observed several months before the strong M7.1 EQ in the Mexican flat slab. At this point, we have to comment that in the case of the Chiapas M8.2 EQ, mentioned above, the variability minimum at the Chiapas area (see Figure 4 of Reference [5]) was accompanied by a simultaneous global minimum in the entire Mexican region (see Figure 2 and Figure 3 of Reference [5]) in accordance with the observations related with the strongest EQ in Japan [65], where the deepest $\beta_{i,min}$ since 1 January 1984 was observed in the first week of January 2011, i.e., approximately two and half months before the 11 March 2011, M9 EQ. An inspection of Figure 2c of Reference [5] that depicts the variability in the entire Mexican region reveals that a shallower local minimum appears during the beginning of 2017.

### 5.3. Identifying the Time of the Impending Mainshock

Here, we apply a procedure analogous to that followed in Reference [5] to estimate the time of the Chiapas M8.2 EQ on 7 September 2017 that has been reviewed in Reference [94]. The criticality relation that has been shown for SES activities [21,22,95] is:

(6) $$\Pi \left(\omega \right)=\frac{18}{5\omega^{2}}-\frac{6\cos\omega }{5\omega^{2}}-\frac{12\sin\omega }{5\omega^{3}},$$

which for ω→0, simplifies to:

(7) $$\Pi \left(\omega \right)\approx 1-0.07\omega^{2}$$

This relation shows, see Equation (1), that $\kappa_{1}$ equals 0.070, which also holds for EQ models, see, e.g., Reference [21].

According to this procedure, that was also followed in References [22,23,58,96,97], the natural time analysis of seismicity in the candidate area starts upon the SES activity initiation. The reason for this choice was based, as mentioned in References [22,65], on the consideration that SES activities are emitted when the focal zone enters the critical stage [69]. Here, we consider the EQs occurring in the flat slab region. In addition, we take advantage of the finding that the appearance of $\beta_{i,min}$ is approximately simultaneous with the SES activity initiation [67]. Hence, here the SES activity initiation should be approximately simultaneous with the $\beta_{i,min}$ computed in the previous subsection, which is around 21 February 2017. Setting natural time zero at the latter date, we form EQ time series in natural time for the flat slab region, each time when a small EQ of magnitude *M* ≥ *M*_thres_ = 3.5 happens; in other words, when the number of events increases by one. The value of Π(ω) for ω→0 (or the variance $\kappa_{1}$) for each of the EQ time series is calculated and compared with that of the above mentioned Equation (6) for ω ∈ [0,π]. The two quantities *S* and *S*_ are also computed.

**Figure 5:**
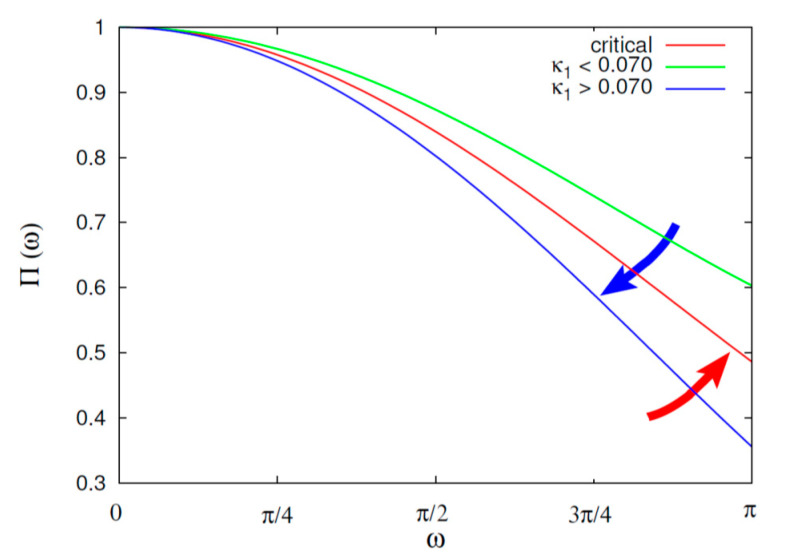
Schematic diagram showing the normalized power spectrum Π(ω) in natural time for ω ∈ [0,π]. The red line is Π(ω) which holds for a critical stage ($\kappa_{1}=0.070$), see Equation (6), whereas the two other lines are for $\kappa_{1}>0.070$ (blue) and $\kappa_{1}<0.070$ (green). The red arrow indicates how the Π(ω) curve approaches the critical from below (the second criterion that should be fulfilled for a true coincidence, see the text).

The criteria to assure a true coincidence of the EQ time series with that of critical state are [21,22,58,96,97]:

(i) The “average” distance 〈D〉 between the curves of Π(ω) of the evolving seismicity and Equation (6) should be 〈D〉 < 10^−2^.
(ii) The final approach of the evolving Π(ω) to that of Equation (6) must be from below as shown by the red arrow in Figure 5 (while the blue arrow indicates the opposite behavior). This reflects that $\kappa_{1}$ gradually changes with time before strong EQs finally approaching from above that of the critical state, i.e., $\kappa_{1}$ = 0.070, as depicted by the inset of Figure 5.
(iii) At the coincidence, both entropies *S* and *S*_ must be smaller than *S_u_*.
(iv) Since this process (critical dynamics) is supposed to be self-similar, the occurrence time of the true coincidence should not vary markedly upon changing the threshold *M*_thres_.

**Figure 6:**
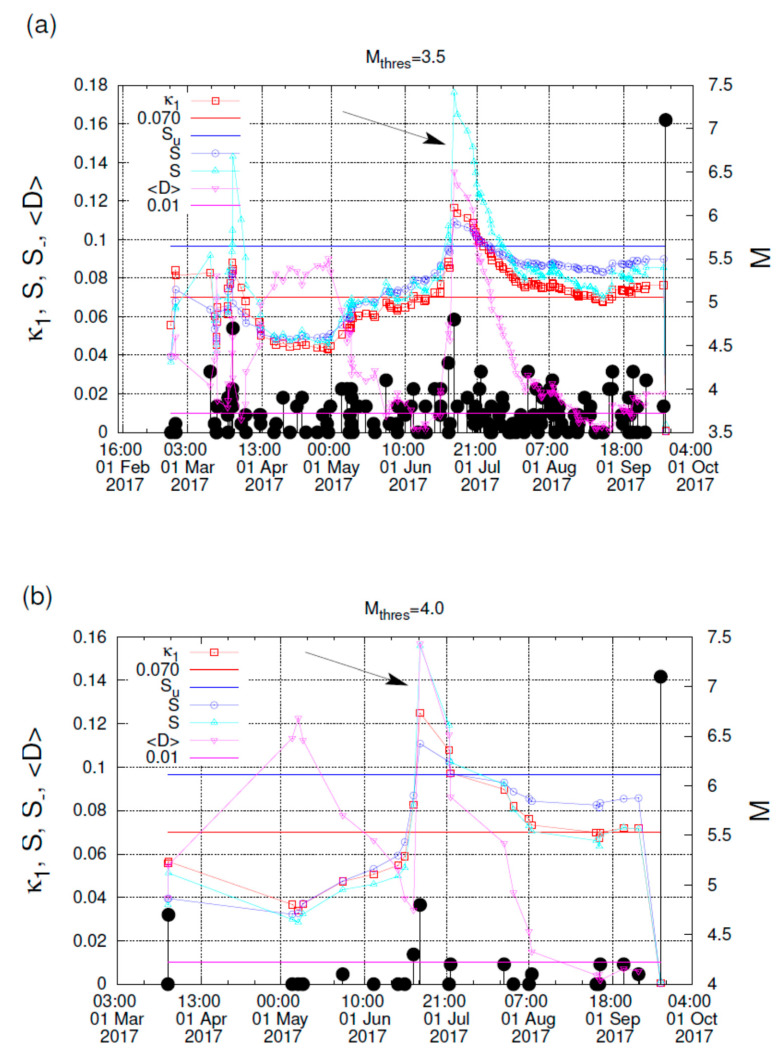
The quantities $\kappa_{1}$, *S*, *S*_ and 〈D〉 computed from the natural time analysis as they evolve versus the conventional time during the period from the beginning of March 2017 until the M7.1 EQ occurrence on 19 September 2017 for (**a**) *M*_thres_ = 3.5 and (**b**) *M*_thres_ = 4.0.

Our results are shown in Figure 6a,b for two different thresholds, i.e., *M*_thres_ = 3.5 and *M*_thres_ = 4.0, respectively. These figures reveal that the above mentioned four criteria are satisfied around the end of August and the beginning of September 2017, thus signaling that the mainshock in the flat slab is going to occur shortly, as actually happened with the occurrence of the M7.1 EQ on 19 September 2017. This result, i.e., satisfaction of all four criteria, is unique during the period after 21 February 2017, which has been obtained on the basis of the variability minimum. The latter, as mentioned in Section 5.2, is also unique during the whole period studied, see Figure 4. On the more general question of the specificity of the variability minima as EQ precursors, one may consult the first paragraph and the ROC diagram in Figure 3 of Reference [93] that has led to an outstanding performance.

A more detailed inspection of Figure 6a,b uncovers the following property: The second criterion for the true coincidence starts to be fulfilled on 21 June 2017. In other words, the quantity $\kappa_{1}$ after 21 June 2017 starts decreasing from values $\kappa_{1}>0.070$ and approaches finally from above the value $\kappa_{1}=0.070$ around the end of August and the beginning of September. In other words, Π(ω) in Figure 5 starts to follow the behavior indicated by the red arrow just after 21 June 2017, i.e., the date of which $\Delta S_{i}\;$ exhibited the minimum observed in Figure 3.

### 5.4. Nowcasting Analysis

We will now apply the nowcasting methodology (see Section 2.2) to the seismicity of the Mexican flat slab. As we said before, we consider all EQs between the isolines of 40 and 60 km Moho depths depicted in Figure 2. Since the smallest magnitude that gives catalogue completeness is 3.5, we take *M* ≥ 3.5, i.e., we have *M_cσ_* = 3.5, and for the large EQs, we choose *M* ≥ *M_cλ_* = 4.7, in order to have a sufficient amount of EQ cycles (cf. this the largest *M_cλ_* for which we have more than 20 EQ cycles, they are actually 25).

The number of EQs in the Mexican flat slab, as already mentioned, is very low compared to other seismic regions linked to Mexican subduction zones. This fact also affects the results obtained in the nowcasting method.

**Figure 7:**
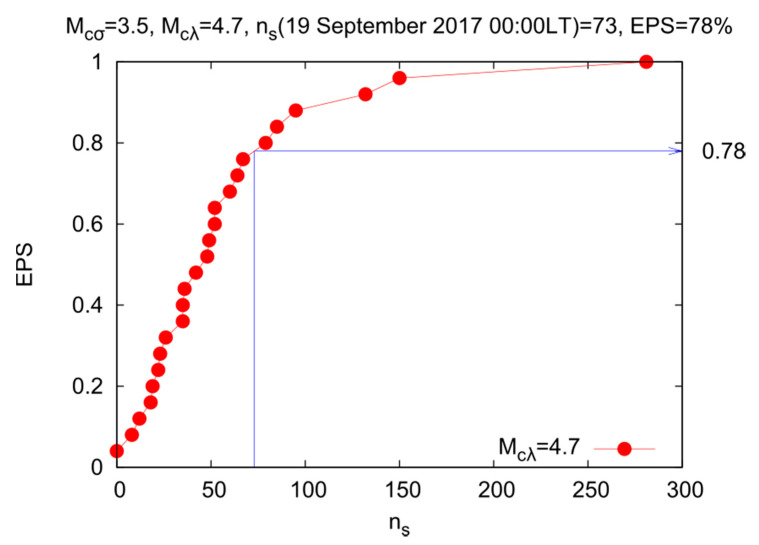
EQ potential score (EPS) for large EQs of magnitude *M* ≥ *M_cλ_* = 4.7, vs. the number *n_s_* of small EQs of magnitude M such that 3.5 ≤ *M* < 4.7 between two large EQs.

The red curve shown in Figure 7 depicts the EPS for the Mexican flat slab. It reveals that when more than *n_s_* = 40 small EQs (4.7 > *M* ≥ 3.5) have occurred, an EQ potential score of around 50% is achieved. Moreover, before the M7.1 EQ on 19 September 2017 one can count that (*n_s_* =) 73 EQs have taken place after the last strong EQ leading to an EPS of 78%. When we take a greater *M_cλ_*, like M5, the number of large EQs becomes very small to apply the nowcasting method.

## 6. Main Conclusions

Since the epicenter of the M7.1 EQ on 19 September 2017 was located in the Mexican flat slab region we analyzed the seismicity (*M* ≥ 3.5) of this region in natural time from 1995 until 2017 and the following conclusions emerged:

The seismicity entropy change Δ*S* under time reversal was found to exhibit a clear minimum on 21 June 2017 upon the occurrence of a M4.8 EQ, almost 3 months before the 19 September 2017, M7.1 EQ. The existence of this minimum is in accordance with the natural time analysis of the OFC EQ model, which is the most studied non-conservative, supposedly SOC model.

It is of major importance that after the appearance of the above Δ*S* minimum, the order parameter of seismicity starts gradually diminishing, thus approaching the critical value $\kappa_{1}$ = 0.070 around the end of August and the beginning of September 2017, which signals that a major EQ is going shortly to occur in the flat slab region.

Moreover, the variability of the order parameter of seismicity shows a minimum during the period February to March 2017.

In addition, the nowcasting method suggested by Turcotte and coworkers was employed here. It revealed that before the M7.1 EQ on 19 September 2017 one can count that (*n_s_* =) 73 EQs have taken place after the last strong EQ on 21 June 2017 leading to an EPS of 78%.
